# Formation of cortical plasticity in older adults following tDCS and motor training

**DOI:** 10.3389/fnagi.2013.00087

**Published:** 2013-12-06

**Authors:** Alicia M. Goodwill, John Reynolds, Robin M. Daly, Dawson J. Kidgell

**Affiliations:** ^1^Centre for Physical Activity and Nutrition Research, Deakin UniversityMelbourne, Australia; ^2^Biostatistics Unit, Faculty of Health, Deakin UniversityMelbourne, Australia

**Keywords:** motor performance, motor training, tDCS, M1 plasticity, inhibition, corticospinal excitability, older adults

## Abstract

Neurodegeneration accompanies the process of natural aging, reducing the ability to perform functional daily activities. Transcranial direct current stimulation (tDCS) alters neuronal excitability and motor performance; however its beneficial effect on the induction of primary motor cortex (M1) plasticity in older adults is unclear. Moreover, little is known as to whether the tDCS electrode arrangement differentially affects M1 plasticity and motor performance in this population. In a double-blinded, cross-over trial, we compared unilateral, bilateral and sham tDCS combined with visuomotor tracking, on M1 plasticity and motor performance of the non-dominant upper limb, immediately post and 30 min following stimulation. We found (a) unilateral and bilateral tDCS decreased tracking error by 12–22% at both time points; with sham decreasing tracking error by 10% at 30 min only, (b) at both time points, motor evoked potentials (MEPs) were facilitated (38–54%) and short-interval intracortical inhibition was released (21–36%) for unilateral and bilateral conditions relative to sham, (c) there were no differences between unilateral and bilateral conditions for any measure. These findings suggest that tDCS modulated elements of M1 plasticity, which improved motor performance irrespective of the electrode arrangement. The results provide preliminary evidence indicating that tDCS is a safe non-invasive tool to preserve or improve neurological function and motor control in older adults.

## INTRODUCTION

Declines in neuromuscular control of the upper limb accompany the process of natural aging. Since many muscles manipulating the wrist insert onto the metacarpals and phalanges, the loss of motor control within the wrist impairs efficiency of hand movements (Armstrong and Chaffin, 1978; Ryu et al., 1991). Such deficits often limit common tasks of daily living, leading to loss of independence (Ward and Frackowiak, 2003). With the projected rise in both life expectancy and retirement age, there is an increasing demand to identify strategies to preserve neuromuscular function with advancing age.

In young adults, acute bouts of motor training modulates primary motor cortex (M1) plasticity in a task-dependent manner (Perez et al., 2004, 2006; Cirillo et al., 2011), with some evidence of improved motor performance (Ziemann et al., 2001; Garry et al., 2004). However, during the aging process, changes in γ-Aminobutyric (GABA) neurotransmission may limit the ability to form use-dependent plasticity following motor training, which potentially limits improvements in motor performance (Sawaki et al., 2003; Fujiyama et al., 2009; Rogasch et al., 2009).

The application of transcranial direct current stimulation (tDCS) represents a promising tool to maintain or enhance motor function in older adults (Hummel et al., 2010; Zimerman and Hummel, 2010; Zimerman et al., 2012). The electrode arrangement appears to be an important factor influencing the physiological outcomes following tDCS; with unilateral tDCS shown to produce either transient increases in corticospinal excitability (anodal) or momentary decreases (cathodal; for review see Nitsche et al., 2008). Additionally, bilateral tDCS has been shown to modulate corticospinal excitability and inhibition within the M1 as well as reduce interhemispheric inhibition (IHI; Lindenberg et al., 2010; Williams et al., 2010; Bolognini et al., 2011; Lefebvre et al., 2012). However, whether these electrode arrangements differentially modulate M1 plasticity and motor performance in older adults is unclear.

Similar to the mechanisms underpinning motor learning, the physiological after-effects of tDCS appear to be associated with long term potentiation (LTP; Liebetanz et al., 2002; Nitsche et al., 2003). Given that older adults experience reduced use-dependent plasticity from motor training alone (Rogasch et al., 2009), the combination of tDCS with motor training may assist to consolidate the mechanisms that are typically observed following motor training alone in younger adults. Currently, only one study in older adults has combined an acute session of unilateral anodal tDCS with motor training, demonstrating improvements in performance up to 24 h following the cessation of stimulation (Zimerman et al., 2012). However, as this study simply measured changes in motor performance, the relationship between tDCS induced M1 plasticity and improvements in motor performance remains unclear.

Experimental evidence in young adults comparing the effects of bilateral and unilateral tDCS on modulating motor performance and corticospinal excitability have produced mixed findings (Vines et al., 2008; Mordillo-Mateos et al., 2012). For instance, Vines et al. (2008) observed greater improvements in motor performance of the non-dominant hand following bilateral tDCS compared to a unilateral and sham condition, whereas Mordillo-Mateos et al. (2012) reported no difference in corticospinal excitability between unilateral and bilateral tDCS. Given that the latter study only examined corticospinal excitability using single pulse TMS, the mechanisms by which tDCS modulates GABAergic inhibitory circuits in older adults remains unknown. Further, neither of the aforementioned studies examined both M1 plasticity and the after-effects on motor performance.

Currently, no studies have investigated the association between tDCS induced M1 plasticity and motor performance in older adults. Further, whether different electrode arrangements differentially modulate motor performance in older adults is unknown. To address these questions, this study compared sham, unilateral and bilateral tDCS combined with motor training on corticospinal excitability, short-interval intracortical inhibition (SICI), and motor performance of the non-dominant distal upper limb in healthy older adults. We hypothesized that unilateral and bilateral tDCS applied during motor training would improve motor performance of the non-dominant limb compared to sham tDCS with motor training alone.

## MATERIALS AND METHODS

### PARTICIPANTS

Eleven healthy older adults (five female, six male; mean ± SEM, 63 ± 2 years; range 55–80) with no history of neurological or musculoskeletal impairment participated in the study. No medications taken by participants influenced central nervous system (CNS) conduction (acimax-1; zometa-1; allopurinol-1; Glucosamine-1; Fish Oil Capsules-1; Minipress-1; Aspirin-1; Karvezide-1; Nexium-1; Oruvail-1). Two participants reported mild arthritis, however this was not confined to the wrist. All participants were tested for handedness to quantify their non-dominant limb, according to the 10 item version of the Edinburgh Handedness Inventory [mean laterality quotient (89.0 ± 5.2)] (Oldfield, 1971). One participant was left handed [mean laterality quotient (-70.0)] and was not excluded from the analyses, rather, this participants non-dominant limb was tested. All participants completed an Adult Safety Screening Questionnaire to determine their suitability for TMS and tDCS application (Keel et al., 2001). Participants were free of any cognitive impairment as assessed by the Mini-mental State Examination (MMSE; mean 29 ± 0.8). All participants completed the long version of the International Physical Activity Questionnaire (IPAQ), consisting of 31 items relating to levels of physical activity, specifically, aerobic exercise (i.e., walking, lifting, running, cycling, and swimming) in a range of areas such as leisure, work, active transport, and household activities ( Fogelholm et al., 2006). No participants reported playing a long term musical instrument. All participants provided written informed consent prior to participation in the study, which was approved by the Deakin University Human Research Ethics Committee. All procedures were conducted according to the standards established by the Declaration of Helsinki.

### EXPERIMENTAL DESIGN

This study was a double-blinded, cross-over sham controlled trial, whereby all participants were exposed to three acute tDCS conditions combined with motor training, with a 1 week washout period between each condition. Active tDCS included both unilateral and bilateral electrode arrangements, whereas the third condition was a sham tDCS. The delivery of each condition was randomized across participants and followed identical testing protocols.

**FIGURE 1:**
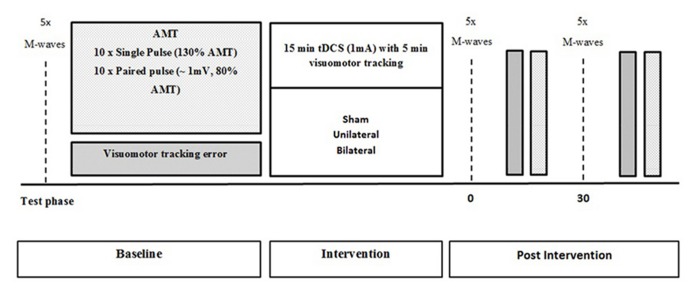
**Schematic representation of the experimental protocol with measures obtained at baseline, immediately after and 30 min after tDCS**.

Participants were required to complete a familiarization session 1 week prior to the commencement of the study to reduce the effect of learning the motor performance task. All participants were tested for baseline measures of corticospinal excitability and intracortical inhibition, as well as motor performance. Following baseline testing, participants were exposed to a total of 15 min of tDCS. After the first 5 min of stimulation, participants performed 5 min of visuomotor tracking. Time-course measures of corticospinal excitability, SICI, and visuomotor tracking error were taken immediately after and 30 min following the stimulation (**Figure 1**).

### TRANSCRANIAL DIRECT CURRENT STIMULATION

Transcranial direct current stimulation was applied over the M1 for 15 min with a fade-in-fade out of 5 s, to avoid alternating currents causing transient neuronal firing. Two 25 cm^2^ electrodes, soaked in a saline solution (0.9% NaCl), were placed over the cortical representation of the extensor carpi radialis longus (ECRL) muscle, as explored and determined with TMS, and secured with a rubber strap. In all conditions, the anode was placed over the non-dominant M1 in the area corresponding with the participants’ non-dominant “ECRL optimal site.” In the unilateral condition, the cathode was placed over the contralateral supraorbital area. During the bilateral condition, the cathode was placed over the dominant M1 corresponding to the “ECRL optimal site.” For the sham condition, participants randomly received unilateral or bilateral electrode arrangements (i.e., 50% of participants were allocated to each arrangement) following the electrode arrangements described above. For all conditions, stimulation intensity was delivered at 1 mA (current density 0.040 mA/cm^2^) through a DC-stimulator (NeuroConn DC stimulator, Ilmenau, Germany). In order to obtain the participants perception of discomfort across the three tDCS conditions, a visual analogue scale (VAS) was used during the first 3 min of stimulation.

### ASSESSMENT OF MOTOR PERFORMANCE

Participants were seated in an office armchair, upright in a neutral position. The elbow was flexed at 90°; shoulder abducted at 45°, and the wrist rested on a chair in the neutral anatomical position. This position allowed free movement of the wrist. Participants were fitted with a sensor icon (i.e., actual limb) driven by a single axis goniometer (3DM-GX2, Williston, VT, USA). Participants were instructed to perform voluntary wrist extension and replicate the movement of a target limb displayed on a PC monitor in front of them, as accurately as possible. The position of the participants wrist joint was displayed as a mirrored anatomical representation of their upper limb, which was positioned parallel to the target limb on the screen. The moving target consisted of three, 30 s unique frames that moved automatically in a vertical manner (i.e., wrist extension and flexion) across the screen with varied frequencies. Each frame was repeated twice, with a total testing time of 3 min.

### MOTOR TRAINING

After the first 5 min of tDCS, all participants performed 5 min of visuomotor tracking. Five, 30 s frames were used with alternating movement frequencies, where each frame was repeated twice.

### RECORDING OF EMG ACTIVITY

Surface electromyography (sEMG) was recorded from the ECRL muscle in both limbs using bipolar Ag-AgCL electrodes. Two electrodes were placed 2 cm apart on the mid belly of the ECRL, with a ground strap placed around the wrist as a common reference for all electrodes. All cables were fastened with tape to prevent movement artifact. The skin was prepared (i.e., shaved and swabbed with alcohol) prior to electrode placement to ensure a clear signal was obtained. sEMG signals were amplified (×100–1000), bandpass filtered (high pass at 13 Hz, low pass at 1000 Hz), digitized online at 2 kHz for 500 ms, recorded and analyzed using PowerLab 4/35 (ADInstruments, Bella Vista, NSW, Australia).

### TMS AND PERIPHERAL NERVE STIMULATION

Single and paired-pulse TMS were delivered over the cortical representation of the ECRL, using a figure-of-eight coil (external wing diameter 90 mm) attached via a BiStim unit, to two Magstim 200^2^ stimulators (Magstim, Dyfed, UK). The coil was positioned over the M1 so that the current flowed in a posterior-anterior direction. Sites near the estimated center of the ECRL were explored to obtain the largest MEP amplitude (i.e., optimal site), and this area was marked by a small “X.” Participants maintained this mark throughout the intervention to ensure consistency and reliability of coil placement within and between sessions.

Measures of corticospinal excitability and intracortical inhibition included active motor threshold (AMT), MEP amplitudes at 130% AMT as well as SICI. All variables were collected at baseline, immediately following and 30 min following the cessation of tDCS. AMT was defined as the stimulator intensity at which at least five out of ten stimuli produced MEP amplitudes of greater than 200 μV. MEP amplitudes were evaluated by producing 10 stimuli at a test-intensity of 130% AMT. All MEPs were recorded during weak voluntary contraction whereby participants positioned their hand in line with their wrist (i.e., anatomically neutral). A constant level of contraction was maintained with reference to an oscilloscope (HAMEG, Mainhausen, Germany) that displayed the root mean square electromyography (rmsEMG) signal (equivalent to 5 ± 2% of maximal rmsEMG activity) in front of the participant. rmsEMG of the ECRL was obtained 100 ms prior to each TMS stimulus.

For the paired-pulse paradigm only, the test-intensity used was the stimulator output required to produce MEPs of ~1 mV. The test-intensity was adjusted if necessary, so that the test MEP amplitudes were always equivalent to ~1 mV (Cirillo et al., 2011). SICI was obtained by delivering a conditioning stimulus at 80% of AMT (subthreshold) followed by a test stimulus (~1 mV; suprathreshold), separated by a 3 ms inter-stimulus interval. Specifically, 10 test stimuli and 10 conditioned stimuli were delivered with the order of presentation randomized throughout the sessions. A rest period of 30 s was provided between stimuli sets to avoid muscular fatigue.

Direct muscle responses (M-waves), were obtained from the ECRL muscle by direct supramaximal electrical stimulation (pulse duration 1 ms) of the radial nerve under resting conditions. A high-voltage constant current stimulator (DS7, Digitimer®, Hertfordshire, UK) delivered each electrical pulse. Stimulation was delivered by positioning bipolar electrodes over the radial nerve on the distal, lateral shaft of the humerus. An increase in current strength was applied until there was no further increase in sEMG amplitude (*M*_MAX_). To ensure maximal responses, the current intensity was increased an additional 20% and the average *M*_MAX_ obtained from five stimuli was delivered and recorded at 0.2 Hz.

All TMS and M-wave procedures were performed for both limbs at each time point and the order of limb testing was randomized across participants and conditions.

### DATA AND STATISTICAL ANALYSIS

Visuomotor tracking error was assessed in 10 s epochs, and calculated by normalizing the root mean square error/deviation by using the actual data’s range (maximum minus minimum) and then converting to a percentage*.* Any MEPs with pre-stimulus rmsEMG that exceeded 5 ± 2% maximal rmsEMG were discarded, and repeated at the appropriate intensity (Sale and Semmler, 2005). *M*_MAX_ and MEP amplitudes were analyzed using LabChart 7.3.6 software (ADInstruments, Bella Vista, NSW, Australia), which provided peak-to-peak values in mV. Single pulse MEPs were normalized to *M*_MAX_ for each individual. In order to quantify SICI, the average conditioned MEP was divided by the average single pulse (i.e., test) MEP and then multiplied by 100. For the variable rmsEMG in the unilateral condition and dominant hemisphere, an outlier was detected and consequently excluded from analysis.

A split-plot in time, repeated measures analysis of variance (ANOVA) was used to determine the effects of motor training with sham, unilateral and bilateral tDCS on all outcome variables (motor performance, MEPs, SICI). One-way ANOVA was used to assess VAS scores across each condition. Paired *t*-tests were conducted to quantify any hemispheric differences in AMT, MEP amplitudes, and SICI. The Greenhouse–Geisser epsilon correction was applied to the degrees of freedom associated with *F*-tests and *t-*tests when Box’s test indicated a departure from the assumption of sphericity and the epsilon was <0.8. Consequently, some *F*-ratios are reported with non-integer degrees of freedom. *F*-tests for main effects and the condition by time interaction were conducted at the 5% significance level. Diagnostic plots of residuals were used to check the assumptions of homogeneity of variance and normality. When significant main effects or interactions were present, Fisher’s LSD was used to compare means. All analyses including calculation of means and SEMs were performed with GenStat statistical software (Release 14.2) using a 5% significance level (*p* < 0.05).

## RESULTS

All participants were comfortable with both TMS and tDCS procedures and reported no adverse side effects. VAS recordings during the first three minutes of tDCS revealed no differences between the perception of the stimulation between conditions, *F*_2,20_ = 0.57, *p* = 0.574; mean ± SEM, 20.6 ± 3.6.

### BASELINE CHARACTERISTICS

**Table 1** displays the mean ± SEM baseline values for measures of corticospinal excitability and intracortical inhibition in both hemispheres. No differences in rmsEMG, *M*_MAX_, AMT, MEP amplitudes, and SICI ratios at baseline were observed across conditions, all *p* > 0.05. There were no hemispheric differences between the dominant and non-dominant M1 for baseline AMT *t*_32_ = -1.88, *p* = 0.070, MEP amplitude, *t*_32_ = -1.16, *p* = 0.255 and SICI ratio, *t*_32_ = 1.54, *p* = 0.134.

**Table 1:** Average ± SEM baseline values for corticospinal excitability and intracortical inhibition in of the non-dominant and dominant M1.

M1	AMT (%)	SI 1_mv_ (%)	*MAX*_(mV)_	Baseline MEP (%*M*_MAX_)	Baseline SICI ratio
ND	30.8 ± 1.8	42.1 ± 2.0	11.5 ± 0.8	14.0 ± 1.6	32.5 ± 1.9
D	29.3 ± 1.6	38.1 ± 1.6	12.6 ± 0.7	13.0 ± 1.8	34.6 ± 2.4

*AMT, active motor threshold; D, dominant primary motor cortex; M1, primary motor cortex; *M*_MAX_, maximum *M*-wave; MEP, motor evoked potential; ND, non-dominant M1; SI, stimulus intensity to produce 1_MV_.*

### rmsEMG

The average ± SEM rmsEMG (μV) prior to single and paired-pulse recordings was 0.053 ± 0.004 and 0.050 ± 0.003, respectively. There were no condition by time interactions for pre stimulus rmsEMG for single pulse, *F*_2.41,36.18_ = 1.40, *p* = 0.261, paired-pulse, *F*_4,60_ = 0.36, *p* = 0.835 signals. For the dominant hemisphere, the average ± SEM pre stimulus rmsEMG for single pulse MEPs was 0.055 ± 0.004 and 0.053 ± 0.004 for paired-pulse MEPs. There was no condition by time interaction for rmsEMG for single pulse, *F*_4,58_ = 1.87, *p* = 0.127, or paired-pulse, *F*_4,60_ = 1.74, *p* = 0.152 signals

### M_MAX_

There was no condition by time interaction for *M*_MAX_ in the non-dominant, *F*_2.63,39.46_ = 0.77, *p* = 0.502, or dominant hemisphere, *F*_4,60_ = 0.18, *p* = 0.947.

### MOTOR PERFORMANCE

**FIGURE 2:**
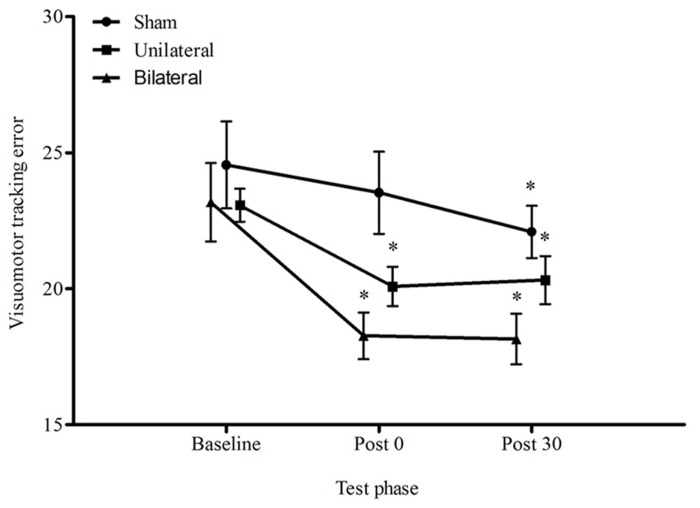
**Average ± SEM tracking error (%) for all conditions across all time points**. ^*^p < 0.05 compared to baseline. No significant condition by time interaction *p* > 0.05.

**Figure 2** displays the average visuomotor tracking errors for each condition across time. There was a significant reduction in the proportion of error over time, *F*_1.38,40.13_ = 19.01, *p* < 0.001 and a significant main effect for condition, *F*_2,19_ = 4.04, *p* = 0.034, however the condition by time interaction was not significant, *F*_2.77,40.13_ = 2.02, *p* = 0.131. Immediately following stimulation, only the unilateral and bilateral conditions improved motor performance by 13%, *p* = 0.006, and 21%, *p* < 0.001, respectively. At 30 min all conditions appeared to improve relative to baseline (sham 10%, *p* = 0.023; unilateral 12%, *p* = 0.012; bilateral 21%, *p* < 0.001). There were no differences between unilateral and bilateral stimulation for either time point, *p* > 0.05.

### MEPs

**FIGURE 3:**
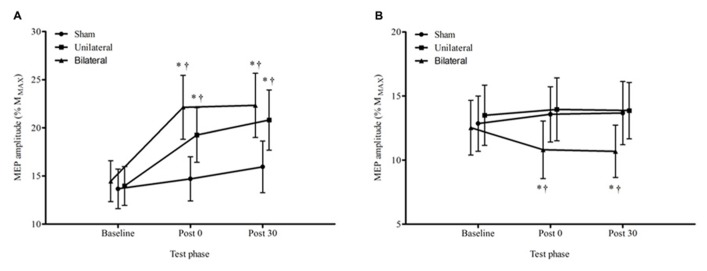
**Average ± SEM MEP amplitudes (% of *M*_MAX_) at 130% AMT for all conditions across all time points. (A)** non dominant M1, **(B)** dominant M1. ^*^p < 0.05 compared to baseline. ^†^*p* < 0.05 compared with sham.

**FIGURE 4:**
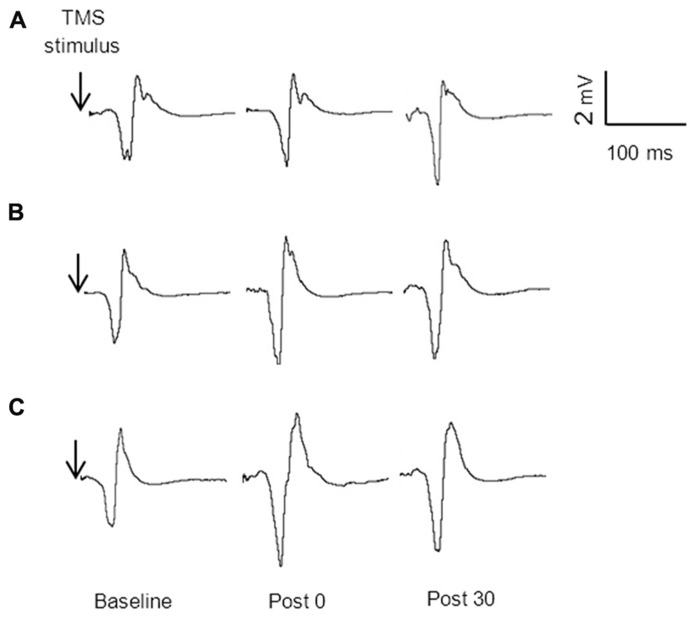
**MEP amplitude (130% AMT) sweeps recorded from one participant at baseline, immediately post and 30 min post stimulation for sham (A) unilateral (B) and bilateral (C) conditions**.

**Figure 3** displays the average MEP amplitudes for each condition across time for the non-dominant hemisphere. There was a significant condition by time interaction *F*_4,60_ = 4.29, *p* = 0.004. The change from baseline to immediately post stimulation for both the unilateral, 38%, *p* = 0.021, and bilateral, 53%, *p* < 0.001, conditions were significantly greater than the change for the sham condition, and this was sustained at 30 min (unilateral 49%, *p* = 0.014, and bilateral, 54%, *p* = 0.003). There were no significant differences in MEP amplitude between unilateral and bilateral conditions at any time point, *p* > 0.05. **Figure 4** provides an illustration of the MEP sweeps recorded at 130% AMT across all conditions and time points for one participant.

For the dominant hemisphere there was a significant condition by time interaction, *F*_4,60_ = 3.74, *p* = 0.009. There were no significant changes, *p* > 0.05, over time in either the sham or the unilateral tDCS condition but in the bilateral tDCS condition the decreases over time from baseline to the immediate and 30 min post time points were significant, 14%, *p* = 0.005 and 15%, *p* = 0.003 respectively.

### SICI

**FIGURE 5:**
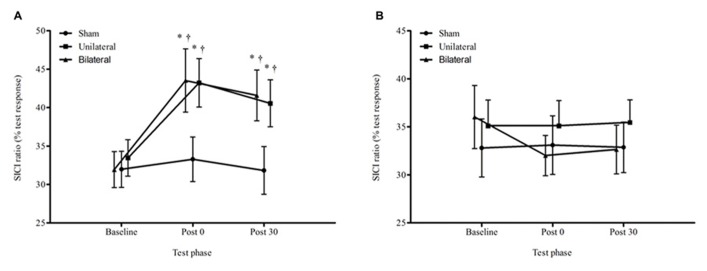
**Average ± SEM SICI ratios (% of the test response) for all conditions across all time points**. **(A)** non dominant M1, **(B)** dominant M1. ^*^p < 0.05 compared to baseline. ^†^*p* < 0.05 compared with sham.

For the average SICI ratios, there was a significant condition by time interaction (*F*_4,60_ = 3.40, *p* = 0.014; **Figure 5**). The change from baseline to immediately post stimulation and 30 minutes post stimulation in the unilateral (post 29%, *p* = 0.013; 30 min 21%, *p* = 0.033) and bilateral (post 36%, *p* = 0.003; 30 min 30%, *p* = 0.005) conditions was significantly greater than for the sham condition (4 and 0.4%). There were no significant differences in SICI ratios between the unilateral and bilateral conditions at any time point (*p* > 0.05).

Finally, for the dominant hemisphere, there was no effect for time, *F*_2,60_ = 1.05, *p* = 0.356 or condition by time interaction for SICI, *F*_4,60_ = 1.41, *p* = 0.243.

## DISCUSSION

The main finding from this study was that motor training combined with unilateral and bilateral tDCS induced M1 plasticity and improved motor performance that persisted for up to 30 min post stimulation, whereas sham improved performance only by 30 min. No differences in the indices of plasticity or motor performance were observed between unilateral and bilateral conditions. Collectively, these findings suggest that combining either unilateral or bilateral tDCS with motor training represents an effective strategy to induce M1 plasticity and expedite motor performance improvements in older adults.

### MOTOR PERFORMANCE FOLLOWING tDCS

Our findings suggest that the tDCS electrode arrangement does not differentially modulate motor performance in older adults. Although motor improvements in older adults have been shown to be sustained following unilateral anodal tDCS (Hummel et al., 2010; Zimerman et al., 2012), a novel aspect to this study was that bilateral stimulation yielded similar improvements in an older population. Although previous findings have suggested a preferential increase in motor performance following bilateral tDCS, compared to unilateral and sham (Vines et al., 2008), this was not evident in the current study. The current findings suggest that the addition of motor training of the non-dominant limb may have augmented the excitatory response from the anode (in both conditions), and thus, modulated corticospinal excitability in a similar manner, contributing to the non-significant differences between unilateral and bilateral conditions.

In this study, the 13–21% improvement in motor performance following active tDCS is slightly larger than a previous investigation in older adults that observed a 2–10% improvement following an acute session of anodal tDCS applied in isolation (i.e., no motor training; Hummel et al., 2010). The greater performance gains in the current study could be explained by the fact that tDCS was applied not only in conjunction with, but prior to a visuomotor training task. It is established that the acute effects of tDCS induce spontaneous neuronal firing which primes the M1 and may consolidate the effects of motor training (Nitsche and Paulus, 2000, 2001; Vines et al., 2006). Although not quantified, the use of a visuomotor task may have activated other cortical and subcortical motor areas that are known contribute to motor performance and learning of a motor task (Bolam et al., 2000). In support of this, the magnitude of performance improvement observed in our study is consistent to the performance improvements induced by visuomotor training in a younger healthy population, without the addition of tDCS (Perez et al., 2006; Cirillo et al., 2011). Given the evidence supporting the reduced effectiveness of motor training alone in older compared with young adults (Rogasch et al., 2009; Zimerman et al., 2012), it appears the addition of tDCS has acted to improve the response to motor training in an older population. This finding suggests that the improved corticospinal activity following tDCS may be beneficial to facilitate improvement in motor performance. However, it should be noted, that interestingly we still observed an improvement in motor performance (10%) at 30 min following motor training alone. In light of this, it seems that the formation of motor performance improvement is facilitated following tDCS when combined with motor training, though, older adults still appear to benefit from motor training alone.

### CORTICOSPINAL EXCITABILITY FOLLOWING tDCS

Motor improvements have been observed following unilateral anodal tDCS in older adults; however the mechanisms underpinning these after-effects have not been previously quantified. Further, no study has examined the effects of bilateral tDCS on modulating corticospinal excitability and motor performance in older adults. The current findings demonstrate no significant differences in the indices of M1 plasticity between unilateral and bilateral tDCS. These results are in agreement with Mordillo-Mateos et al. (2012) who also observed that corticospinal excitability in young adults was not differentially modulated by unilateral and bilateral tDCS. Given these findings, we speculate that the alternate tDCS electrode arrangements induce similar physiological mechanisms, underlying the improvements in motor performance.

Our findings show that in older adults, both unilateral and bilateral tDCS facilitated MEPs both immediately after and 30 min following stimulation. These findings are in agreement with studies performed in young adults whereby MEPs were increased following an acute bout of either unilateral or bilateral tDCS (Nitsche and Paulus, 2000, 2001; Lang et al., 2004; Williams et al., 2010; Mordillo-Mateos et al., 2012; Kidgell et al., 2013). In the dominant M1, MEP amplitudes were suppressed for the bilateral condition only, which supports previous literature in young adults (Williams et al., 2010). Reduced MEPs within the dominant M1 suggests that the cathode may have suppressed motor overflow of neuronal activity in the M1 ipsilateral to the trained limb (dominant M1), however this did not appear to differentially affect motor performance. In light of this finding, there were no differences in corticospinal excitability or motor performance of the non-dominant limb following either unilateral or bilateral tDCS suggesting the effect of the cathode over the dominant M1 may have been minimal. Although we observed a 15% reduction in corticospinal excitability of the dominant M1, previous data has suggested that individual variability in the response to tDCS, in particular cathodal stimulation, may be due to genetic factors such as the polymorphism of brain-derived neurotrophic factor (BDNF; Antal et al., 2010). Although not quantified in this study, the potential for these factors to contribute to the non-significant differences in between the responses to unilateral or bilateral tDCS cannot be overlooked. Therefore, it should be considered that there may be no optimal electrode arrangement for inducing plasticity and improving motor performance in this population.

The finding of tDCS induced corticospinal excitability outlasting the stimulation period, appears reflective of LTP-like mechanisms that have been observed following motor learning in rats (Sanes and Donoghue, 2000; Rioult-Pedotti et al., 2000), and has more recently have been suggested to occur in humans (Ziemann et al., 2004). Previous pharmaceutical investigations have applied dextromethorphane (an NMDA receptor antagonist) to alter the after-effects of tDCS (Liebetanz et al., 2002; Nitsche et al., 2003), therefore it can be proposed that the after-effects of tDCS are indicative of modifications in NMDA receptor dependent neurotransmission. The small and non-significant increase in corticospinal excitability following motor training with sham tDCS, supports the reduced ability for older adults to form use-dependant plasticity following motor training alone (Sawaki et al., 2003; Rogasch et al., 2009). The current results speculate that during natural aging, there may be a limited response to plasticity inducing protocols that reflect the involvement of LTP-like processes. *In vivo* studies have certainly shown that in an aging rat model, the interaction between dopamine, GABA and glutamate in the basal ganglia is decreased, which may be reflective of decreased activity in glutamate receptor binding sites (i.e., NMDA receptor; Segovia and Mora, 2005; Mora et al., 2008). Additionally, in aged human brain tissue, a reduction in both dopamine uptake and NMDA receptor activity have been observed (Villares and Stavale, 2001). Given that these neurotransmitters located in the basal ganglia are important for the acquisition and performance of motor patterns, degeneration of these structures may contribute to the delayed onset of motor performance improvement observed in this study. Collectively, the current evidence suggests the additive combination of tDCS and motor training may have improved sensitivity and unmasking of excitatory synapses at the post-synaptic membrane, improving synaptic efficacy, and neural transmission along the corticospinal pathway (Nielsen and Cohen, 2008).

### INTRACORTICAL INHIBITION FOLLOWING tDCS

It is established that GABA dependent neurotransmission plays an important role in shaping excitatory output, and is partially modulated by NMDA receptor activity (Ziemann et al., 1998, 2001; Zoghi et al., 2003). Therefore, the balance of GABA mediated intracortical inhibition is vital for efficient and coordinated movement (Rothwell et al., 2009; Marneweck et al., 2011). Previously, the effect of tDCS on intrinsic inhibitory circuits has not been investigated in older adults. The current study demonstrated a release of SICI in the non-dominant M1, following both unilateral and bilateral tDCS relative to sham, but importantly, there were no differences between the two active tDCS conditions.

The current finding that tDCS reduced SICI by 21–36% is comparable to studies in young adults and post stroke patients, which demonstrates that improvements in corticospinal excitability may be modulated by reduced intracortical inhibition (Hummel et al., 2005; Nitsche et al., 2005; Edwards et al., 2009; Batsikadze et al., 2013). Contrary to our findings, Williams et al. (2010) combined bilateral tDCS with motor training in healthy young adults and found no effect on SICI. Although comparisons to this study should be viewed with caution (due to different tDCS parameters used), the age of the cohort used in the current study may have contributed to the tDCS induced changes in SICI. Certainly, there is evidence for age-related deficits in SICI circuitry (Sale and Semmler, 2005; Fujiyama et al., 2009), and therefore it is possible that older adults may respond more favorably to tDCS.

Based upon the current findings it can be speculated that the reduction in GABAergic inhibition has improved the synaptic efficacy between intracortical and corticospinal neurons (Nitsche et al., 2003). In support of this, previous data in the rat M1 suggests that synaptic plasticity is enhanced by a release of intracortical inhibition (Hess et al., 1996). Interestingly, there was a non-significant change in SICI in the dominant M1, possibly contributing to the non-significant differences between the release of SICI following the two active tDCS conditions. Although, a recent study in an aging population observed differences in spatial activation between bilateral and unilateral tDCS (Lindenberg et al., 2013), it is conceivable that contribution from other motor control pathways such as interhemispheric networks, basal ganglia circuits and the posterior cingulate cortex involving GABAergic synapses may contribute to corticospinal excitability and motor performance improvements following tDCS (Bolam et al., 2000; Baudewig et al., 2001; Williams et al., 2010; Lindenberg et al., 2013). Irrespective of this, our results support the contribution of released GABA-related inhibitory activity in M1, on the overall net excitatory output and improved motor performance of the non-dominant limb in older adults.

### LIMITATIONS

Several limitations of the study need to be considered. The hypothesis that bilateral tDCS may induce greater motor performance gains was based around hemispheric differences during aging. Although there was a larger percentage improvement for performance following bilateral tDCS, the non-significant differences between unilateral and bilateral tDCS may be due to a lack of hemispheric differences in the cohort of healthy older adults recruited into this study. Further, we used a conditioning stimulus of 80% AMT which has been shown to induce SICI mediated by GABA_A_ receptors (Zoghi and Nordstrom, 2007) however, the interaction between intracortical inhibitory and facilitatory circuits contributing to the reduction in SICI should be considered when interpreting these findings (Shirota et al., 2010). Lastly, the small sample size may have not been powerful enough to detect significant differences between unilateral and bilateral stimulation in this population.

## CONCLUSION

In conclusion, this study indicates that tDCS induced M1 plasticity and an expedited improvement of motor performance in older adults, irrespective of the electrode arrangement. These findings underscore the prospective use of tDCS to improve the activity of neurons within the M1 and motor performance in the elderly. As repeated bouts of tDCS have been suggested to have a cumulative effect (Boggio et al., 2007; Reis et al., 2009), future investigations need to provide larger sample sizes and longer trials assessing retention, to evaluate whether the tDCS electrode montage differentially improves motor performance in this population.

## Conflict of Interest Statement

The authors state that there are no actual or potential conflicts of interest associatedwith the research. Study participants providedwritten informed consent, and the protocol was approved by the Deakin University Human Research Ethics Committee.

## AUTHOR CONTRIBUTIONS

All experiments were conducted in the Neuroscience laboratory at Deakin University, Burwood, VIC, Australia. Alicia M. Goodwill was involved in the conception and design of the experiment as well as collecting, analyzing, and interpreting the data. Alicia M. Goodwill was also involved with the drafting and critical revision of the article. Dawson J. Kidgell was involved in the conception and design of the experiment, interpretation of data and critical revision of the article. Robin M. Daly was involved in the conception and design of the experiment, analyses and interpretation of the data and critical revision of the article. John Reynolds was involved in analysis and interpretation of the data as well as critical revision of the article.
